# A Novel Deep Learning Model for Human Skeleton Estimation Using FMCW Radar †

**DOI:** 10.3390/s25133909

**Published:** 2025-06-23

**Authors:** Parma Hadi Rantelinggi, Xintong Shi, Mondher Bouazizi, Tomoaki Ohtsuki

**Affiliations:** 1 Graduate School of Science and Technology, Keio University, Yokohama 223-8522, Japan; 2 Department of Computer Engineering, Universitas Papua, Manokwari 98314, Indonesia; 3 Faculty of Science and Technology, Keio University, Yokohama 223-8522, Japan

**Keywords:** FMCW radar, human motion analysis, multi-head attention, point cloud, skeleton detection

## Abstract

Human skeleton estimation using Frequency-Modulated Continuous Wave (FMCW) radar is a promising approach for privacy-preserving motion analysis. However, the existing methods struggle with sparse radar point cloud data, leading to inaccuracies in joint localization. To address this challenge, we propose a novel deep learning framework integrating convolutional neural networks (CNNs), multi-head transformers, and Bi-LSTM networks to enhance spatiotemporal feature representations. Our approach introduces a frame concatenation strategy that improves data quality before processing through the neural network pipeline. Experimental evaluations on the MARS dataset demonstrate that our model outperforms conventional methods by significantly reducing estimation errors, achieving a mean absolute error (MAE) of 1.77 cm and a root mean squared error (RMSE) of 2.92 cm while maintaining computational efficiency.

## 1. Introduction

Millimeter wave (mmWave)-based sensing has gained wide attention in recent decades due to its ability to detect humans with high sensitivity and precision [1]. Moreover, using mmWave radar has many advantages over camera systems because it can operate without requiring lighting and without compromising privacy, thus allowing for more continuous and safe monitoring [2].

Recent developments in mmWave technology, including hardware such as Frequency-Modulated Continuous Wave (FMCW) radars, offer novel opportunities in the field of human sensing. Tasks such as human action recognition and body motion require new solutions in signal processing technology and deep learning models to improve sensing capabilities.

The development of mmWave technology to estimate human joint positions in patient rehabilitation systems [3] has advantages over video-based methods because it uses point cloud data, not only preserving the privacy of the monitored person but also offering depth information that cannot be extracted from a typical video. This technology also enables detecting and locating human joints and monitoring the human body in a multitude of scenarios, such as during sleep [4,5].

Liu et al. [6] proposed a method that supports mmWave technology in capturing the human joints. Their method relies on a neural network model that employs an entire attention mechanism for skeleton-based action recognition. Moreover, convolutional neural network (CNN)-based approaches can improve the accuracy of human skeleton estimation [1,7]. In addition, the point-convolution model approach can improve the extraction of local information and point cloud density [8] for accurate human skeleton pose estimation.

Furthermore, mmWave radar-based human skeleton estimation research has shown significant improvements, leading to essential studies such as [9], which applied a sequence-to-sequence (Seq2Seq) model commonly applied in Natural Language Processing (NLP) to process sequences of point cloud data from radar and generate 3D position estimates of human skeleton key points.

In addition to CNNs, researchers have employed other deep learning techniques for human skeleton estimation. In particular, Long Short-Term Memory (LSTM) [10] has shown to be very suitable in capturing long-term dependencies in sequential data, making it ideal for tasks such as sequence prediction and classification.

Another deep learning technology that improves the performance of human skeleton estimation is transformer, which has a multi-head attention mechanism capable of extracting high-level features [11]. This technology, when combined with LSTM, can better capture temporal dependencies.

However, the main challenge arises when the mmWave point cloud data used is very sparse and carries little information compared to other data formats, such as data collected using cameras or Light Detection And Ranging (LiDAR) devices. To overcome this problem, a multi-frame representation fusion model was employed in [12]. However, the mean absolute error (MAE) value of the obtained human skeleton estimation is still high.

To solve this problem, we propose a new framework to estimate the positions of 19 human joints that combines adjacent frames into a new one and design a neural network to capture the spatiotemporal data in this new frame. Concatenating adjacent frames helps to improve the quality of human skeleton-related data points, where merging these frames is an initial phase before entering the main processing stage, which involves CNN, transformer, and Bi-LSTM layers put together in a deep learning neural network for human skeleton estimation. This paper is an extended version of our previous work [13], where we first introduced the concept of frame concatenation and an attention-based CNN–LSTM model for joint estimation. In this version, we provide a more detailed explanation of the methodology, conduct more extensive experiments, and evaluate the model’s performance on unseen subjects and activities.

The contributions of this paper can be summarized as follows:

1. We introduce a novel mmWave-based deep learning model that accurately detects human joints, allowing to recognize and analyze skeletal movements. Our model builds upon the existing methods by offering enhanced spatiotemporal feature extraction, leading to clear improvements in detection accuracy and computational efficiency in human skeleton estimation.
2. We propose a new preprocessing method that combines adjacent frames into a single one, which is then processed by our main deep learning model that estimates human joint positions.
3. We validate the proposed approach on a public dataset to demonstrate its effectiveness in estimating human joint positions. The model’s performance is compared to the existing approaches [1,3] to highlight its advantages and limitations. The outcomes emphasize the model’s strengths in achieving higher accuracy and efficiency for radar-based human skeleton estimation.

The remainder of this paper is as follows: Section 2 provides an overview of the existing works on human skeleton estimation using mmWave radar data. In Section 3, we introduce our proposed framework. Section 4 introduces the experimental specifications in detail and presents and discusses the results. Finally, in Section 5, we conclude our work, providing a clear roadmap for future work.

## 2. Related Work

In recent years, mmWave radar has become increasingly popular thanks to its ability to operate in dark environmental conditions. It is known for its wavelength, which is in the millimeter range, and mmWave radars operate by transmitting high-frequency electromagnetic waves (e.g., 77–80.2 GHz [3,14]) and analyzing the reflected signals to detect objects and measure their distance, velocity, and angle. It can also capture fine-grained movements such as human joint motion, respiratory movements, and small limb displacements, which are essential for applications like pose estimation and healthcare monitoring. This is achieved through techniques like FMCW modulation, which allows precise range and velocity measurements by analyzing the frequency shift and phase differences in the reflected signals. The ability of this radar to combine several transmit and receive antennas makes the mmWave radar more precise in detection.

One of the research topics involving mmWave radar is pose estimation, where radar point clouds provide a spatial representation of human motion relative to the radar’s field of view. Using radar point cloud data to estimate the position of human joints in 3D in [3] is very profitable because it is cheaper, overcomes privacy issues, and does not depend on lighting, which affects other devices such as cameras. Moreover, the multi-frame representation process can improve radar point cloud data and accelerate model adaptation [12]. Nevertheless, combining features output from different deep learning models was proven to improve the pose estimation accuracy [15]. Some valuable techniques like sorting spatial axes and merging points from frames proved to be useful as well [16].

Sengupta et al. [17] proposed a CNN-based pose estimation approach using mmWave radar data that creates two 3D feature vectors from each radar data point, the first using depth, azimuth, and intensity and the second using depth, height, and intensity. Moreover, the study treats each vector as an image pixel, stacking all the pixels from all the radar points in a frame into an array of size (16 × 16) to create two arrays of images with size (16 × 16 × 3), and processes them using a CNN. Another proposal in [9] introduced a seq2seq model for pose estimation by changing the radar points into voxels. The seq2seq approach is used to process the data through an encoder block and predict the voxelized pose key points at the output iteratively. At the final stage, the pose key locations will be obtained. Another study used voxelization to detect sitting direction using sequential modeling [18].

Another related topic in which mmWave radars were explored is human activity recognition (HAR). The main challenge in developing HAR systems using mmWave radar lies in the limitations and difficulties of collecting extensive, clean, and labeled experimental data. Research using a simulation-based approach in [19] developed an end-to-end simulation framework capable of simulating a realistic mmWave radar based on human motion. Other research using mmWave radar for HAR proposed a pre-learning model trained using 3D coordinates from radar data paired with Kinect data as the ground truth [20]. This model scheme generates more reliable estimates of human joint coordinates in 3D from sparse radar data. Once reliable features are extracted, a Graph Neural Network (GNN) model is used to identify human activities based on predicted joint coordinates.

Another work that uses the Graph Convolutional Network (GCN) approach in frame-based action recognition does not rely on a predefined graph structure but dynamically computes the graph structure based on input movement patterns [6]. Then, a spatial attention module aims to capture spatial features from other nodes at the same timestep, while a temporal attention module aggregates temporal features in the local temporal environment. In [21], Ahmad et al. used a combination of CNN- and Bidirectional Gated Recurrent Unit (Bi-GRU)-generated features with feature selection for human activity recognition techniques using deep-temporal learning. Meanwhile, a deep learning-based method [22] involved a new approach in human pose estimation using mmWave waves and the Inverse Synthetic Aperture Radar (ISAR) algorithm, where the ISAR algorithm produced high-resolution radar images of moving human targets. The main contribution of the research is the development of a robust ISAR-Pose pipeline for radar-based human pose estimation, which can function in various environmental conditions and can be used for both individual and multi-person pose estimation.

In [23], Stevens et al. discussed the development of a joint estimation model that leverages knowledge of the human body kinematics to enhance sensor accuracy. They used an experimental approach with two 3D CNNs trained to estimate the coordinates (x,y,z) of 19 body joints based on point cloud data generated by mmWave sensors. Other research works such as [24,25], which used mmWave data, relied on advanced kinematics to overcome noise artifacts.

Neural networks like CNNs have been widely used to process human activity recognition data [26]. Moreover, an approach combining a CNN and LSTM for detecting human joints was implemented in [27], which can achieve competitive estimation accuracy with low MAE values. Another model [10] applied neural network structures such as CNN, LSTM, and ResNet to improve the performance of deep learning models in non-contact human motion detection applications. In this paper, we also use a CNN to extract features from the concatenation of adjacent frames before entering the next processing stage, namely transformer.

Other works applied a transformer to HAR, where the model is designed to work with mmWave point cloud sequences to address the unique characteristics of point cloud data, such as irregularity and sensitivity to environmental noise [28,29]. Another work [30] used a hybrid learning architecture that combines a temporal transformer and LSTM to track joint positions based on global and local information of joint trajectories.

The above works motivated us to use the concatenation of adjacent frames followed by the use of three neural network blocks, namely CNN, transformer, and Bi-LSTM, to improve the tracking and detection of 19 human joint positions.

The role of the transformer in our research is to take the output of the CNN sub-network and process it in the multi-head attention layer to capture spatial and temporal relationships. The output of the transformer, in turn, will be processed by a Bi-LSTM network producing the predictions of the positions of the 19 human joints.

## 3. Proposed Methodology

This section introduces the proposed model and the principle of concatenating adjacent frames, which we blend into the main model. The model incorporates a CNN sub-network, a multi-head transformer block, and a Bi-LSTM block, offering a new perspective regarding human joint detection.

**Figure 1:**
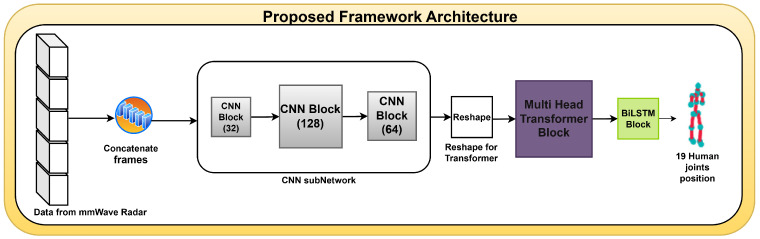
The framework of the proposed method for human joint detection.

The proposed model architecture for detecting human joints involves concatenation of frames, where consecutive frames are combined into one new more informative frame. Figure 1 illustrates the proposed model architecture for detecting human joints, showcasing the different steps of our approach.

### 3.1. Concatenate Adjacent Frames

**Figure 2:**
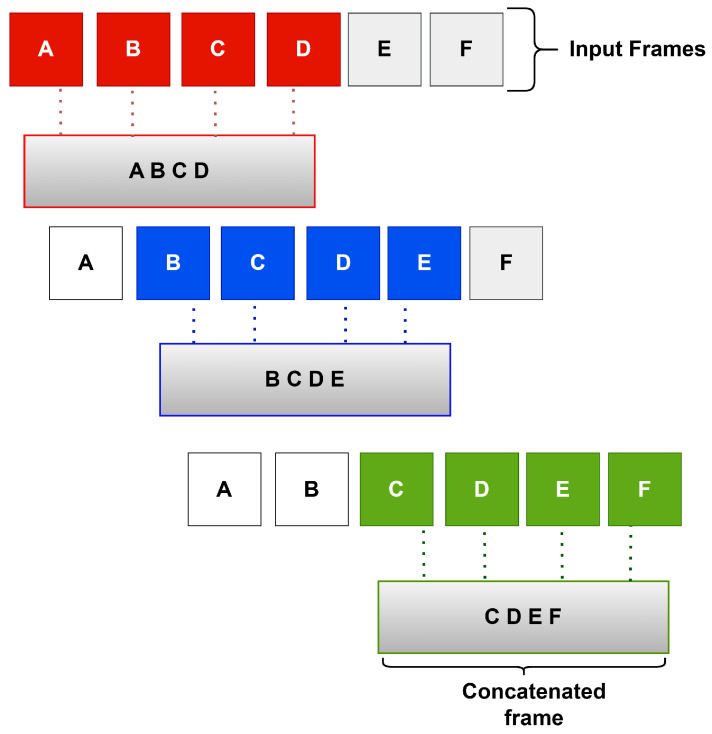
An example of concatenation of adjacent frames.

The concatenation step can be described as follows. Suppose that we have 6 consecutive frames: A, B, C, D, E, and F. Each time, *n* adjacent frames are combined into a single frame. For example, if *n* is equal to 4, frames A, B, C, and D are combined into one new frame, namely the frame ABCD. In a similar way, the frames B, C, D, and E are combined into the new frame BCDE. Finally, the frames C, D, E, and F are combined into one new frame, namely CDEF. This stage ends up producing fewer frames but with larger dimensions so that the resulting frames can be used by our neural network model. Figure 2 illustrates the process of frame concatenation.

**Figure 3:**
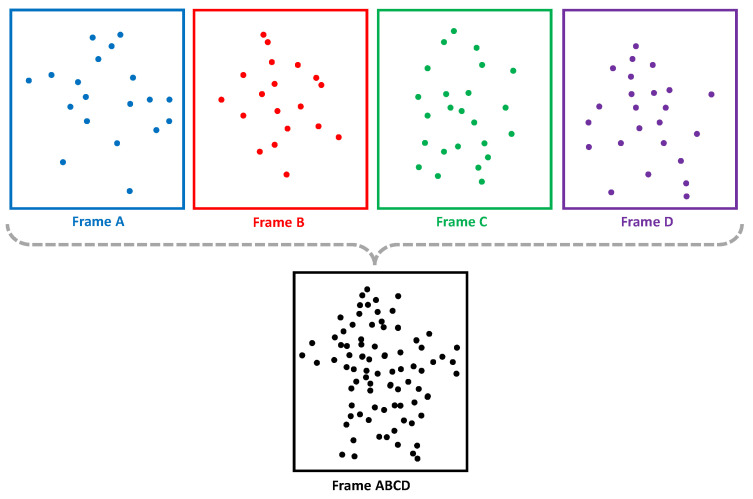
An example concatenation in point cloud domain for temporal processing.

To construct an enriched temporal representation, adjacent frames are concatenated sequentially along the temporal axis rather than the spatial axis. This ensures that the spatial structure of each frame remains intact while capturing the dynamic evolution of movement. Unlike a 3D CNN, which processes spatial correlations across consecutive frames using volumetric convolutions, our model employs a transformer block to model long-term dependencies and a Bi-LSTM layer to extract temporal correlations. Figure 3 shows an illustration of how multiple consecutive point cloud frames are concatenated along the temporal axis. Each frame (Frame A, Frame B, Frame C, and Frame D) retains its original spatial structure. When combined, the resulting point cloud is much denser. Being within a short time window from each other allows us to perform the concatenation. A long time window can lead to detail loss of individual frames, whereas a short time window results in a non-dense point cloud. Therefore, the choice regarding the appropriate number of frames to concatenate is important, as we will demonstrate later.

Compared to the multi-frame representation fusion approach introduced in [12], where features are extracted independently from each frame and fused at the feature level, our method applies early fusion by concatenating raw adjacent frames before any feature extraction. This enables the network to learn joint spatiotemporal representations directly from the input and reduces redundant computation, resulting in a more efficient and coherent modeling process.

Let $X^{\left(i\right)}$ be a sample representing a sequence of frames from the dataset with dimensions

(1) $$X^{\left(i\right)}\in R^{T\times H\times W\times C}$$

where *T* is the number of frames in the sequence, *H* and *W* represent the spatial dimensions of each frame, and *C* is the number of channels in the input. For each frame index *j*, the adjacent frame concatenation is performed by concatenating four consecutive frames:

(2) $$Z_{j}^{\left(i\right)}=\mathrm{concat}\left(X_{j}^{\left(i\right)},X_{j+1}^{\left(i\right)},X_{j+2}^{\left(i\right)},X_{j+3}^{\left(i\right)}\right)\in R^{4C}$$

where j=1,2,...,T−3, yielding a transformed representation:

(3) $$Z^{\left(i\right)}\in R^{(T-3)\times H\times W\times 4C}$$

### 3.2. Integration to the Main Model

**Figure 4:**
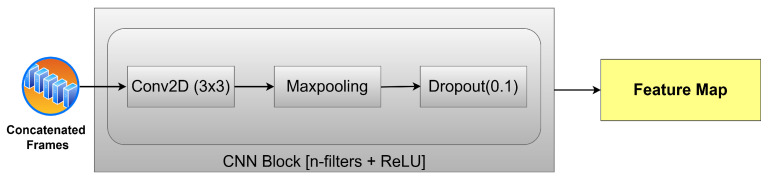
The details of CNN layers.

The frames produced by the concatenation phase are then processed by a series of convolutional neural network layers, as shown in Figure 1, consisting of three CNN layers using 32, 128, and 64 filters, respectively, as shown in Figure 4; each filter has a kernel size of 3 × 3. The convolutional layers have an ReLU activation each, followed by MaxPooling that takes the maximum value of 2 × 2. Next, a dropout layer is used with a rate of 0.1. The function of this series of convolution layers is to extract essential features from the data in each frame and generate feature maps, which are then processed further by the transformer layer.

Here is the mathematical equation to extract features from the concatenated $Z^{\left(i\right)}$ using the Time-Distributed CNN architecture, where each frame in the sequence is processed using a separate 2D convolution. This operation can be formulated as follows:

(4) $$Z_{t}^{\left(i\right)}=\sigma \left(W^{\left(1\right)}*Z_{t}^{\left(i\right)}+b^{\left(1\right)}\right),\;t=1,\ldots ,\left(T-3\right)$$

where * is the 2D convolution operation, and σ(·) is the ReLU activation function. After passing through several convolution layers with MaxPooling and dropout, the representation of the extracted features is expressed as the following equation:

(5) $$F^{\left(i\right)}\in R^{\left(T-3\right)\times H^{\prime }\times W^{\prime }\times C^{\prime }}$$

where $H^{\prime },W^{\prime }$ are the feature sizes after the downsampling process by MaxPooling; $C^{\prime }$ is the number of feature channels after the convolution layer. Equations (4) and (5) directly represent the feature map after going through the CNN block shown in Figure 4.

Although the convolutional layers operate on spatial information, the input tensor of concatenated adjacent frames along the temporal axis implicitly contains temporal transitions between frames. This structure enables the CNN to capture short-term temporal correlations as spatial patterns. For example, gradual changes in joint positions across adjacent frames appear as structured differences that CNN can learn through its filters. Therefore, while CNN does not explicitly model temporal dependencies, its latent feature space reflects motion-induced correlations across the concatenated frames. Explicit temporal modeling is handled subsequently by the transformer and Bi-LSTM layers.

A reshape layer is then employed to change the shape of the tensor without changing the data itself. This layer is used to prepare the output of the convolution layer to match the input required by the transformer layer. Before reshaping, the final convolutional layer output has the shape [bach size, number of frames, height, width, number of channels]. For instance, in our experiments, the batch size is 128, and the dimensions are therefore [128, 18, 28, 14, 5]. Here, the number of frames is 18, the spatial dimensions are 28 and 14, and 5 is the number of channels. The reshape function transforms this input into [128, 18, 28 × 14 × 5], keeping the dimensions “batch size” and “number of frames” and fusing the remaining dimensions into one. The final form is [128, 18, 1960], where each time frame is represented as a feature vector of size 1960. It is important to transform spatial data into a series of feature vectors that the transformer can handle.

Next, this feature is reshaped so that it can be processed by the transformer layer, as shown in Equation (6) below:

(6) $$F^{\left(i\right)}\in R^{(T-3)\times D},\;D=H^{\prime }\times W^{\prime }\times C^{\prime }$$

This approach allows the model to capture spatial relationships within each frame while preserving temporal information through concatenation of adjacent frames and CNN.

**Figure 5:**
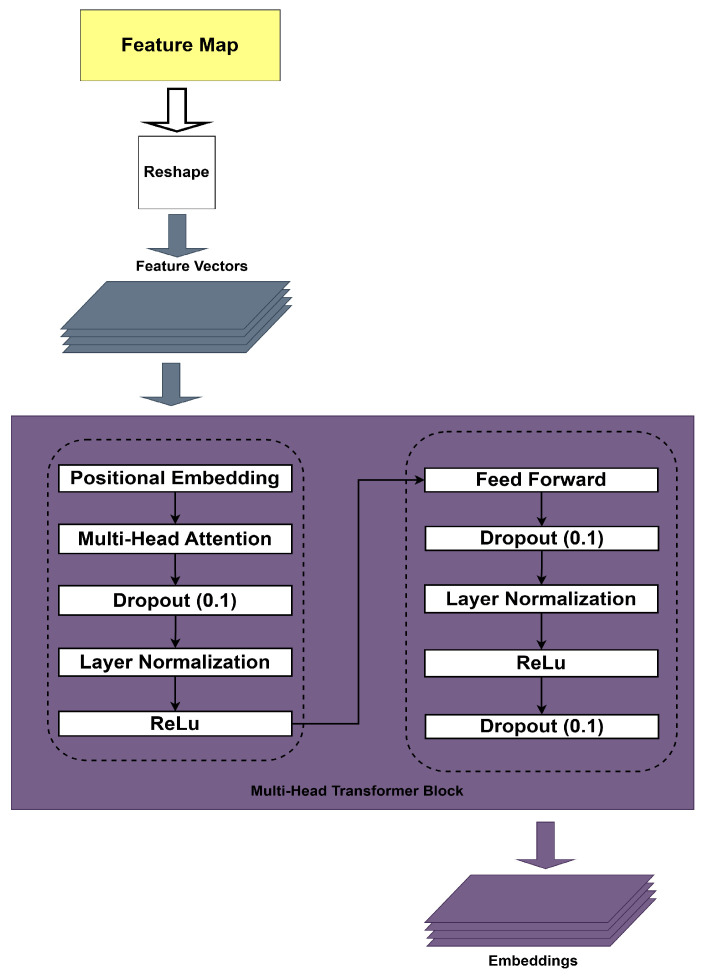
The details of the multi-head transformer block.

The reshaped data is fed to the multi-head transformer block, as shown in Figure 5. This data goes through the positional embedding stage, a technique used in the transformer model to provide information about the sequence or position of each element in the input sequence. This is important because transformers have no built-in understanding of data order.

Multi-head attention lets the model focus on different parts of the input sequence simultaneously. This layer has eight attention units. Figure 5 shows that this block includes three dropout layers with a drop rate equal to 0.1 to help prevent overfitting, and two normalization layers to normalize the input to stabilize training and speed up convergence. In the multi-head transformer block, an ReLU layer is used to add non-linearity, which is essential for capturing complex relationships in the data. In addition, a Feed-Forward Network (FFN) processes each time frame independently. Regarding transformer processing stage equation, first of all, positional encoding is added to the extracted features as follows:

(7) $$E_{t}^{\left(i\right)}=F_{t}^{\left(i\right)}+P_{t},\;P_{t}=\mathrm{Embedding}\left(t,D\right)$$

After that, the multi-head attention (MHA) layer is applied to capture the temporal relationship between frames as in Equation (8):

(8) $$A_{t}=\mathrm{MHA}\left(E_{t}^{\left(i\right)},E_{t}^{\left(i\right)},E_{t}^{\left(i\right)}\right)$$

This process is formulated in the following Equation (9) with a multi-head attention mechanism.

(9) $$\mathrm{MHA}\left(Q,K,V\right)=\mathrm{softmax}\left(\frac{QK^{T}}{\sqrt{d_{k}}}\right)V$$

Then, Layer Normalization and Feed-Forward Network (FFN) are applied:

(10) $$X_{t}^{\left(i\right)}=\mathrm{LayerNorm}\left(E_{t}^{\left(i\right)}+A_{t}\right)$$

(11) $$X_{t}^{\left(i\right)}=\mathrm{ReLU}\left(W^{\left(2\right)}X_{t}^{\left(i\right)}+b^{\left(2\right)}\right)$$

The results of this stage will be used for further processing in Bi-LSTM to capture temporal information more effectively.

**Figure 6:**
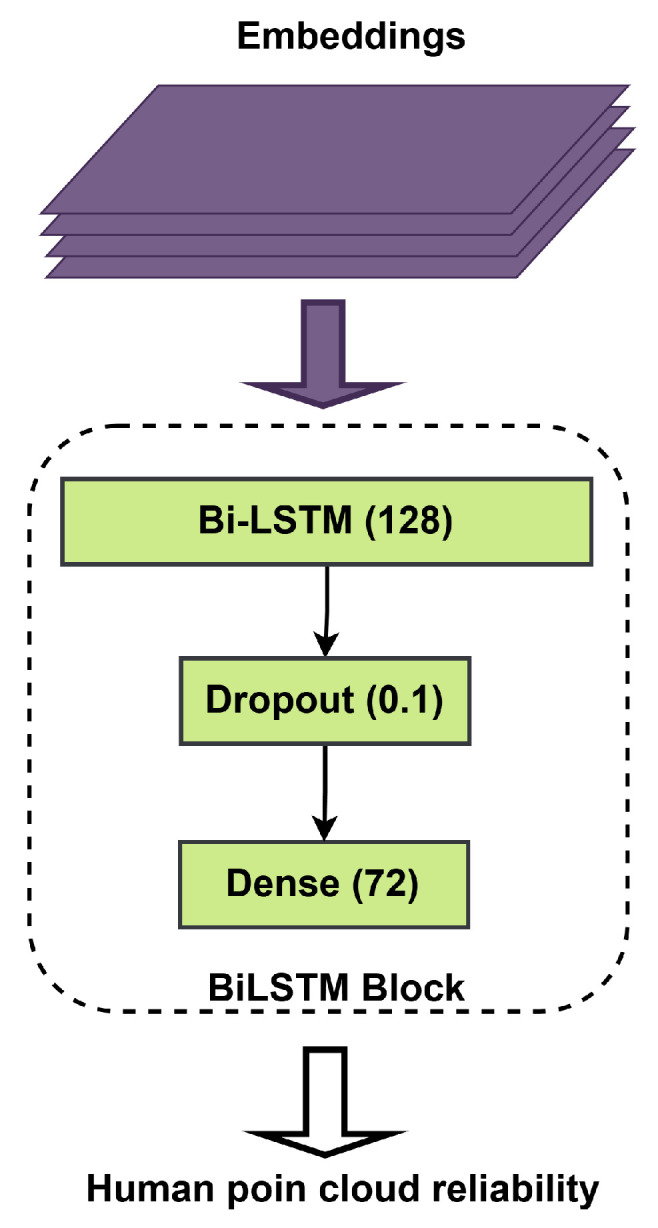
The details of the Bi-LSTM layers.

In the next step, Bi-LSTM uses the multi-head transformer block output. Figure 6 explains the Bi-LSTM block, which aims to read a two-way input data sequence. This is very important for predicting the position of critical points with high accuracy. In our model, the number of units in the LSTM was set to 128, and two dropout layers whose drop rate is set to 0.1 were employed to prevent overfitting. In addition, a dense layer with an output vector of dimension 72 is used to predict 19 human joint points. The neural network architecture used in this work is shown in Table 1. The formulation of this operation is provided as follows:

(12) $$h_{t}=\mathrm{BiLSTM}\left(X_{t}^{\left(i\right)}\right)$$

where

(13) $$h_{t}=\mathrm{LSTM}\left(X_{t}^{\left(i\right)}\right)⊕{\mathrm{LSTM}}_{\mathrm{reverse}}\left(X_{t}^{\left(i\right)}\right)$$

The Bi-LSTM concatenates the hidden states from both forward and backward directions, yielding the final hidden representation:

(14) $$H^{\left(i\right)}\in R^{2d},\;d=128$$

where *d* represents the dimensionality of the LSTM hidden states in each direction. This representation is then passed to the fully connected layers for keypoint regression. The output from the Bi-LSTM layer is then passed through fully connected layers to generate the final prediction. The first fully connected layer applies a non-linear transformation:

(15) $$y^{\left(i\right)}=\mathrm{ReLU}\left(W^{\left(3\right)}H^{\left(i\right)}+b^{\left(3\right)}\right)$$

where $W^{\left(3\right)}\in R^{d\times d^{\prime }}$ and $b^{\left(3\right)}\in R^{d^{\prime }}$ are the weight matrix and bias, respectively. A dropout layer is applied to prevent overfitting:

(16) $$y^{\left(i\right)}=\mathrm{Dropout}\left(y^{\left(i\right)}\right)$$

Finally, the output layer predicts the keypoints using a linear transformation:

(17) $${\hat{y}}^{\left(i\right)}=W^{\left(4\right)}y^{\left(i\right)}+b^{\left(4\right)}$$

where $W^{\left(4\right)}\in R^{d^{\prime }\times K}$ and $b^{\left(4\right)}\in R^{K}$ are the weight matrix and bias of the final layer, respectively. The output ${\hat{y}}^{\left(i\right)}$ represents the estimated keypoint positions.

## 4. Experiments and Results

This section presents the experiment methods, the results, and a discussion of the proposed model.

### 4.1. Experimental Setup

In this research, we followed the division of training, validation, and testing sets as in the original paper [3], using their public dataset MARS [3], which is designed to assist in rehabilitating patients with motor disorders using the mmWave radar system. This dataset consists of 10 types of rehabilitation movements performed by four human subjects, with a total of 40,083 frames. This dataset includes the reconstruction of 19 human body joints in 3D space using a point cloud generated by the mmWave radar. The activities performed include left arm extension, right arm extension, both arm extension, left front lunge, right front lunge, squat, left side lunge, right side lunge, left leg extension, and right leg extension. Each subject performed each movement for 2 min, resulting in approximately 10,000 data frames per subject.

This data was then used for model training, validation, and testing. Data collection was conducted indoors to ensure lighting and radar signal reflection consistency. Specific conditions considered during the experiment included no strict limitations as mmWave radars are independent of lighting conditions. The subject stood at 2 m from the radar. The radar was mounted on a 1-meter-high table with a Microsoft Kinect V2 sensor (Microsoft Corp., Redmond, WA, USA). Reference data was obtained from the Kinect V2, which recorded the subject’s movements at a rate of 30 Hz.

Variability in mmWave radar data affects detection accuracy due to several factors. Noise in radar data arises from signal interference, environmental materials, and impacting the generated point cloud. Occlusion occurs when body parts are obscured due to natural movements, such as arms covering the chest during squats or variations in hand positions affecting radar reflections. Additionally, sparsity in point cloud data makes mmWave radar less dense than camera-based or LiDAR methods, necessitating the concatenation of adjacent frames to enhance spatial–temporal information before processing with CNN, transformer, and Bi-LSTM. Furthermore, inter-individual movement variability, influenced by differences in height, posture, and execution of rehabilitation movements, affects data distribution. To improve generalization, the model is trained on data from multiple subjects.

The experiments were conducted using the TI IWR1443 76-81GHz model radar (Texas Instruments, Dallas, TX, USA) is available at (https://www.ti.com/product/IWR1443 (accessed on 8 June 2024)), which produces high-quality point clouds. The radar technical details are provided in Table 2. As shown in this table, the radar configuration includes a frequency range of 76–81 GHz, 3 transmitting antennas, and 4 receiving antennas (resulting in 12 virtual channels), a chirp bandwidth of 3.2 GHz, and a chirp duration of 32 μs. This setup enables a range resolution of 4.69 cm, a velocity resolution of 0.35 m/s, and an angular resolution of approximately 9.55°, which collectively contribute to the generation of high-quality point clouds used for human joint estimation. For the human skeleton estimation task, we initially employed an optimal number of 19 frames [1]. The radar technical details in Table 2 include the hardware and software configuration used in this experiment.

All experiments in this research were conducted using carefully selected parameters, as detailed in Table 3. These parameters can be used for reproducibility of our results.

As explained in Section 3.1, this research implements an approach that combines several adjacent frames into a single more informative one. This method enhances the quality of the data before it is processed in the main blocks of the detection neural network.

After going through several experiment stages, the results were evaluated using the metrics mean absolute error (MAE) and root mean squared error (RMSE) measured for the *x*, *y*, and *z* coordinates of 19 joints. Moreover, the average MAE and RMSE values of the *x*, *y*, and *z* axes are calculated to provide overall performance information. Furthermore, the computational complexity associated with the model can be derived from the number of parameters trained during model training.

### 4.2. Results

As detailed in Section 4.1, the experiment began with 19 frames, as suggested in [1]. The initial shape of the point cloud before entering the frame merging stage is [128, 19, 14, 14, 5], with 19 representing the initial frames, 14 × 14 the point cloud data, and 5 the channel. The dataset then undergoes the adjacent frame concatenation process, a key step before being trained in the three main blocks: CNN, transformer, and Bi-LSTM. Table 4 displays the experimental results of four different configurations. These configurations represent the same network for different numbers of concatenated frames, referred to as n=1, n=2, n=3, n=4, and n=5.

As explained, we start with 19 frames for each instance, which undergo the concatenation process in which multiple consecutive frames are combined into a single one. To determine the best choice, five different experiments were carried out as shown in Table 4. Here, n=1, represents a configuration without any frames concatenated. n=2 represents combining two adjacent frames into one new frame with the shape [128, 18, 28, 14, 5], where 18 is the number of frames, 28 × 14 is feature map, and 5 is the depth. n=3 represents combining three adjacent frames, which results in an output with the form [128, 17, 42, 14, 5]. Similarly, n=4 represents the combination of four adjacent frames into one, and n=5 represents the combination of five adjacent frames into one. Their respective outputs are [128, 16, 56, 14, 5] and [128, 15, 70, 14, 5].

The results of observations on the different configurations, as shown in Table 5, show that n=1 and n=2 have the worst performance because they are less capable of capturing complex features when compared to models with more parameters. n=3 and n=5 are slightly better even though there is still the potential for overfitting. This overfitting arises from the increase in the number of parameters and the limitations of the data. The increase in parameters occurs because, as more frames are combined, the model has to learn more parameters. This makes it easier for the model to memorize the training data rather than learning generalized patterns. Furthermore, a data limitation arises because the dataset size is not large enough to balance the increase in parameters, causing the model to adapt to specific patterns in the training data. As a result, the model performs well on the training data but fails on new data. n=4 has the best results among all the models because it provides a balance in capturing spatial and temporal patterns, making the model the best choice for estimating human joint position in scenarios that require high precision. As a result, n=4 is treated as the optimal configuration in this research.

In addition to empirical validation, the choice of n=4 is theoretically supported by the temporal resolution of the radar system. The MARS radar operates at 30 Hz, meaning each frame captures approximately 33 milliseconds of motion. By concatenating four consecutive frames, the network observes around 133 ms of temporal context, sufficient to capture micro-motion such as limb lifts or subtle joint displacements. Furthermore, since the dataset uses overlapping windows to generate training samples, increasing *n* beyond 4 introduces substantial redundancy. For example, with n=5, each sample shares four out of five frames with the previous one (80% overlap), reducing sample diversity and leading to higher inter-sample correlation. This makes it more difficult for the model to generalize and learn robust spatiotemporal features. Therefore, n=4 is not only optimal empirically but also theoretically justified based on radar sampling characteristics and the dataset construction strategy.

Table 6 shows the MAE and RMSE values from the experimental results of n=4 to detect the positions of 19 human joints. The MAE and RMSE values of all 19 joint points are in the range of 1~4 cm, with the exception being the left and right wrists. The average MAE values are 1.94, 1.62, and 1.76 cm for the *x*, *y*, and *z* axes, respectively. Similarly, the average RMSE values for the *x*, *y*, and *z* axes are 3.28, 2.39, and 3.07 cm, respectively. This outperforms, by a large margin, the the method proposed in [3] and noticeably outperforms the method proposed in [1]. The average joint values are smaller than 4 cm. Moreover, our experimental results can reduce the MAE and RMSE values in the right and left wrist joints, where previous research in [3] was unable to detect them accurately.

As previously indicated, the architecture of our model (for n=4 as well as other configurations) is given in Table 1. Our neural network was designed to capture patterns both locally and globally. Local pattern identification refers to the process of identifying spatial and short-term temporal relationships in the data. This process is conducted via the CNN and transformer blocks. The global pattern identification refers to the process of identifying long-term temporal relationships in the data (between non-adjacent frames) via the LSTM sub-network.

**Figure 7:**
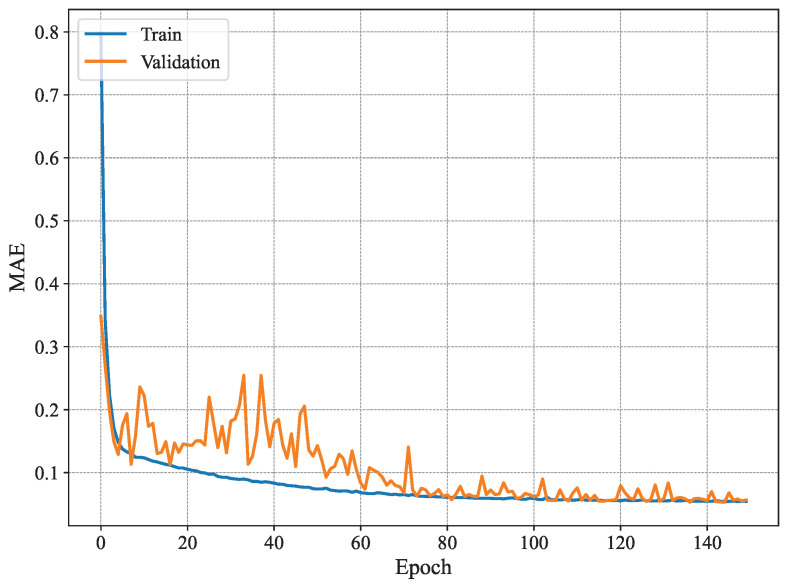
Average MAE of 19 joint estimates on training and validation sets using MARS.

**Figure 8:**
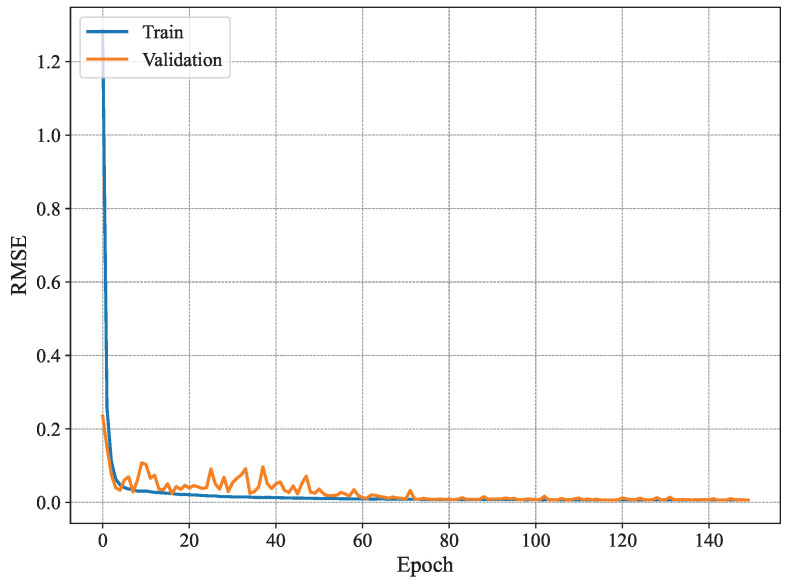
Average RMSE of 19 joint estimates on training and validation sets using MARS.

Figure 7 shows the MAE per epoch for the method proposed in [3]. The results show that the training MAE starts at high values, as expected, and decreases gradually with every epoch. However, this behavior is not observed for the validation set, where the MAE does not seem to drop significantly until later epochs (i.e., after 80 epochs). Nonetheless, it fluctuates remarkably even for later epochs. Similar behavior is observed for the RMSE shown in Figure 8, where the training RMSE decreases consistently, whereas the validation one fluctuates until around epoch 70. This behavior may be attributed to the high number of trainable parameters and the architecture of the model proposed in [3], which likely contributes to overfitting, whereby the model learns specific patterns in the training set that do not generalize to unseen data, such as the validation data.

**Figure 9:**
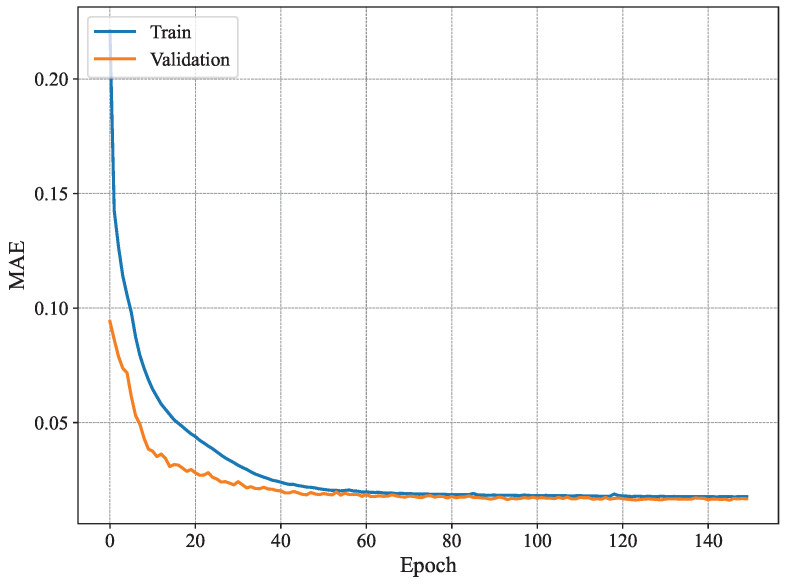
Identification MAE for n=4.

**Figure 10:**
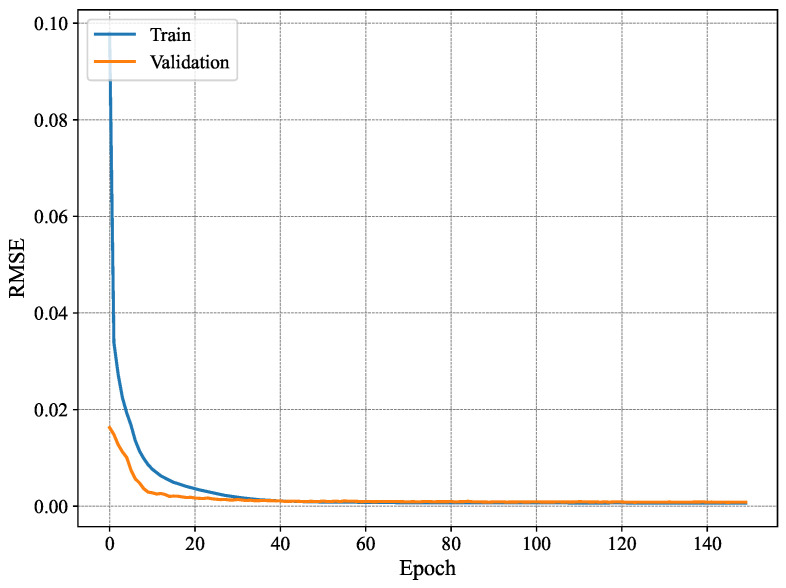
Identification RMSE for n=4.

Figure 9 shows the MAE per epoch for our proposed method using n=4. As can be seen, our model is more stable than the conventional method [3] and exhibits typical good learning behavior where both the training and validation estimation MAE decrease consistently to converge towards similar values. Starting from epoch 60 or so, the model seems to have converged, and the MAE remains within the same value range until the end of the training. Compared to the results presented in Figure 7, the training behavior of our system model is much better as it does not exhibit any signs of overfitting, namely the fluctuations in validation estimation MAE and the difference in performance between the training and validation curves. Furthermore, Figure 10 shows the RMSE of our proposed model with n=4. The results show similar patterns to those of the MAE, where the model converges slowly until it reaches good performance around epoch 60. The increase in stability is a key difference between the model proposed in [3] and our model for n=4 of our proposed model. There has been a reduction in overfitting, and model generalization has increased. Our model offers a good option for handling temporal data and reducing fluctuations often occurring in validation data. Our model is more effective for time-dependent data, such as radar data, for human joint detection.

Next, the experimental results of n=4 are compared with the conventional methods [1,3,15,17]. Table 7 and Table 8 show the estimation accuracy of human joint locations using these five models (i.e., ours and the conventional ones). The model in our experiment shows significant improvement in estimation accuracy when compared with [3]. Our proposed model reduces the MAE by 4.1 cm, which indicates a substantial increase in model performance. This can be attributed to the concatenation of adjacent frames, which allowed producing new frames that are more informative. Furthermore, the layers of the transformer block preserve spatiotemporal information, transforming into comprehensive features for the Bi-LSTM to predict the positions of the different joints with high accuracy. This research produces a robust model for the task of human skeleton estimation. Our research model effectively reduces estimation errors and proves to be better in handling frames.

We performed statistical tests to evaluate the significance of the MAE and RMSE values in the proposed and conventional models. We employed paired *t*-tests and *p*-values and examined both. We compared the two values for the proposed model with those of the conventional method. Table 9 shows the results of the *t*-tests and the *p*-values for MAE.

The proposed model demonstrates significant improvement compared to MARS [3] and mmPose [17] (*p* < 0.05), indicating that this model is statistically more accurate in estimating joint positions than those methods. There is no significant difference compared to CNN+Bi-LSTM [1] (*p* > 0.05), which indicates that the performance of this model is comparable to CNN+Bi-LSTM. The improvement compared to mRI [15] is almost significant (*p* ≈0.07), which means the proposed model is likely better. However, the statistical test is not enough to confirm this difference with certainty. Table 10 shows the *t*-test results and *p*-values for RMSE on the conventional model for comparison with the proposed model.

The most significant difference was seen in mmPose [17] (*p* = 0.019), indicating that the proposed model is statistically superior in reducing joint position estimation errors compared to mmPose [17]. This model is also better than MARS [3] (*p* = 0.038), indicating that this difference is statistically significant (*p* < 0.05). The improvement compared to CNN+Bi-LSTM [1] (*p* = 0.194) and mRI [15] (*p* = 0.075) is not significant, which means that, although the RMSE of this model is lower, the difference is not statistically strong enough to conclude an absolute advantage over these methods.

We also include confidence intervals (CIs) for the reported metrics to indicate the reliability of the results. Based on the experimental results of 95% CIs for MAE and RMSE, we can analyze how stable and reliable the proposed model is compared to the baseline method. Table 11 shows the CI results of the conventional and proposed models.

The proposed model has the best performance because it has the lowest MAE and RMSE and the smallest CI. The CNN+Bi-LSTM model [1] is quite close to the performance of the proposed model but still has higher variability. Moreover, MARS [3] and mmPose [17] have very high variability, showing inconsistent and less reliable results.

Next, a detailed comparison of inference time was conducted to determine the computational efficiency. In the context of neural networks such as CNN, LSTM, or transformer, inference time refers to the duration from when data enters the model until the model produces a result. The experiments contain accuracy and computational costs that will be used for analysis. Table 12 compares the inference time and accuracy in the proposed and conventional models.

Based on the comparison results in Table 12, it can be concluded that the proposed model has the highest accuracy of 98.77%, outperforming CNN+BiLSTM [1] and MARS [3]. However, this increase in accuracy must be paid for with a longer inference time, which is 0.28 ms per sample, compared to CNN + BiLSTM [1] and MARS [3]. MARS [3] and CNN+BiLSTM [1] have low inference times due to their simpler architecture. The proposed model has the longest inference time due to the complexity of the architecture, such as the implementation of a transformer, multiple layers, and concatenating adjacent frames, which increases computation.

To demonstrate the generalizability of the data, the proposed model is tested on unseen activities. In the next step, we implement the Leave One Subject Out (LOSO) method in experiments to test the model in unseen subjects to show generalization outside the dataset [3]. Four subjects participated in our experiments. Therefore, to appropriately implement LOSO, we run four rounds. During each of them, three subjects’ data are used for training, and the remaining data are used for testing. This aims to measure the generalizability of the model to new subjects. In this test, we employed the proposed model and conventional models such as MARS [3] and CNN+Bi-LSTM [1]. Table 13 shows the experimental results regarding MARS [3] for the LOSO method.

The performance of the MARS model [3] has relatively high errors, both in terms of MAE and RMSE. The highest overall average error was found when testing Subj_2. MARS models [3] tend to be less able to generalize to untrained subjects, resulting in significant errors.

Table 14 shows the accuracy of the CNN + Bi-LSTM model [1] regarding unseen subjects in the LOSO framework. The performance of this model is significantly improved compared to MARS [3]. The highest error occurred when testing Subj_2 and the lowest when testing Subj_3. CNN+Bi-LSTM [1] shows better temporal and spatial sequence learning ability than MARS [3] but still has room for improvement in accurate estimation in all three axes (x,y,z).

Table 15 shows the evaluation of the proposed model (*n* = 4) using the LOSO protocol for the joint estimation. This model shows the best results compared to the other two models. The average MAE is 2.06–4.12 cm, and the RMSE is 2.46–5.90 cm. The best results were obtained when testing Subj_2. The proposed model can capture spatial and temporal relationships very well and shows strong generalization to previously unseen subjects. The small error values demonstrate the superiority of this model architecture in handling mmWave radar data for joint point estimation.

In addition, we evaluated unseen activities with the Leave Some Activities Out (LSAO) method regarding the proposed approach. LSAO aims to test the model’s generalization ability to activities not seen during training. We split the dataset into five subsets, where each subset includes some activities exclusively. We then proceeded to train different models using different subsets. We run four rounds, where, in each round, four of the five subsets are used for training, and the remaining one is used for testing. By performing five rounds, five models are created on some activities and evaluated on others. This allows us to properly evaluate the proposed method on unseen activities. Table 16 shows the experimental results using the LSAO method on the proposed model. The average MAE and RMSE were stable and low overall, indicating that the model generalized well to unseen activities (i.e., activities that were not encountered during training). Low MAE and RMSE values for the *x*-axis mean the model is very stable in recognizing horizontal changes. The *y*-axis (depth) shows the highest MAE and RMSE in all rounds. This is common in mmWave radars as the depth axis is often more prone to distortion or low resolution in estimation. Furthermore, dynamic body orientation towards the sensor (depth) and lack of variation in activity data on the *y* axis during training further degrade the detection along this axis. For the *z*-axis, performance is more stable and consistent. Round 3 appears to have the most representative and generalizable combination of testing activities from the training data. It can be seen that, in some rounds, the model is able to adapt well to unseen activities.

Table 17 provides a detailed ablation study based on the MAE and RMSE values for joint estimation across the *x*, *y*, and *z* axes and the average over all the axes. The analysis reveals the individual contribution of each architectural component, CNN, transformer, and Bi-LSTM, toward the model’s spatial estimation accuracy.

The proposed model, which includes all three components (CNN + transformer + Bi-LSTM), achieves the best overall performance with the lowest average error (MAE: 1.77 cm; RMSE: 2.92 cm). When a GRU replaces the Bi-LSTM, the average error increases slightly to 1.84 cm MAE, suggesting that Bi-LSTM provides stronger temporal modeling capabilities, although GRU remains a reasonable alternative.

Removing the transformer leads to further degradation (MAE: 2.07 cm), indicating its critical role in capturing temporal dependencies via attention mechanisms. Removing the Bi-LSTM entirely yields a similar drop (MAE: 2.30 cm), reaffirming the importance of sequential modeling. In contrast, removing the CNN results in the highest overall error (MAE: 3.66 cm; RMSE: 5.76 cm) due to the model’s inability to extract meaningful spatial features early on.

Axis-specific trends show that the *x*- and *z*-axes produce lower errors. At the same time, the *y*-axis often has slightly higher values across configurations, possibly reflecting the radar’s lower depth resolution. These results confirm that each component contributes complementarily to the spatial and temporal learning process and justify the architectural choices made in the proposed model.

**Figure 11:**
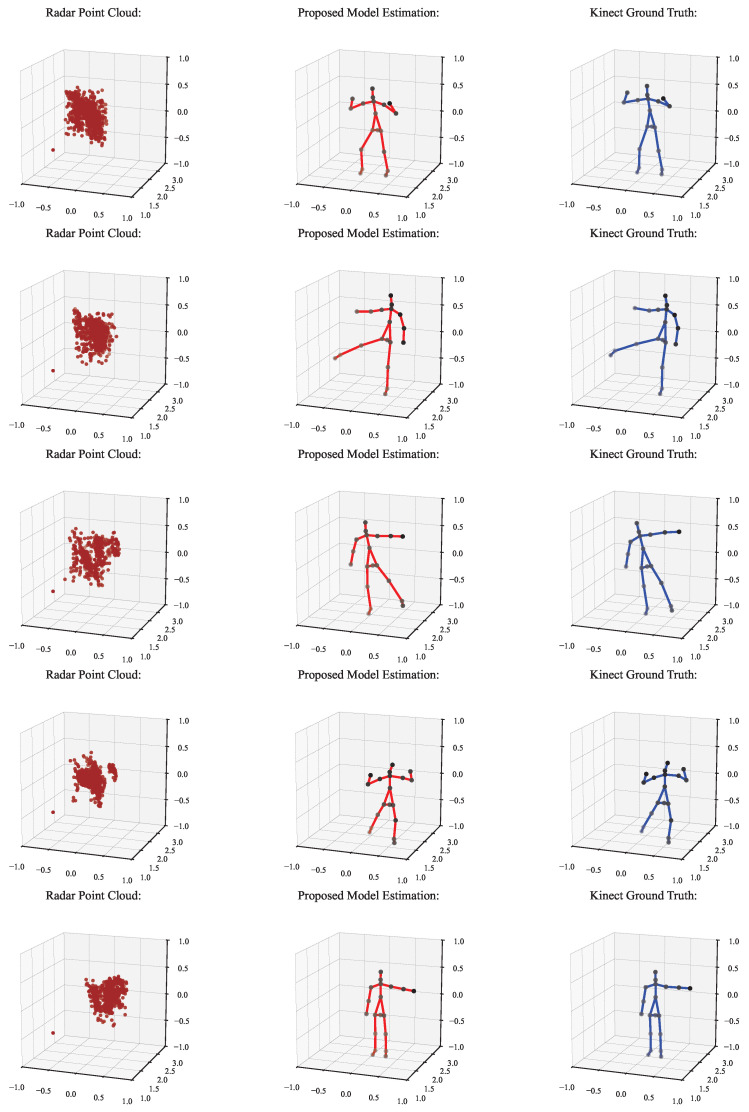
Visual comparison of radar point cloud, predicted skeleton by the proposed model, and Kinect-based ground truth over multiple frames.

Figure 11 illustrates the qualitative results of the proposed skeleton estimation framework. Each row in the figure corresponds to a representative frame sampled from a sequence. From left to right, the subfigures show (a) the input radar point cloud, (b) the predicted human skeleton obtained from our model, and (c) the ground-truth skeleton derived from the Kinect sensor. The visual comparison shows that the predicted joints align closely with the reference joint locations in different poses, indicating that the model can learn accurate spatial representations from the sparse radar data. This visual evidence complements the quantitative results in Table 6, Table 7 and Table 8 and confirms the robustness of our method in capturing complex limb configurations, such as lunges and extensions.

**Figure 12:**
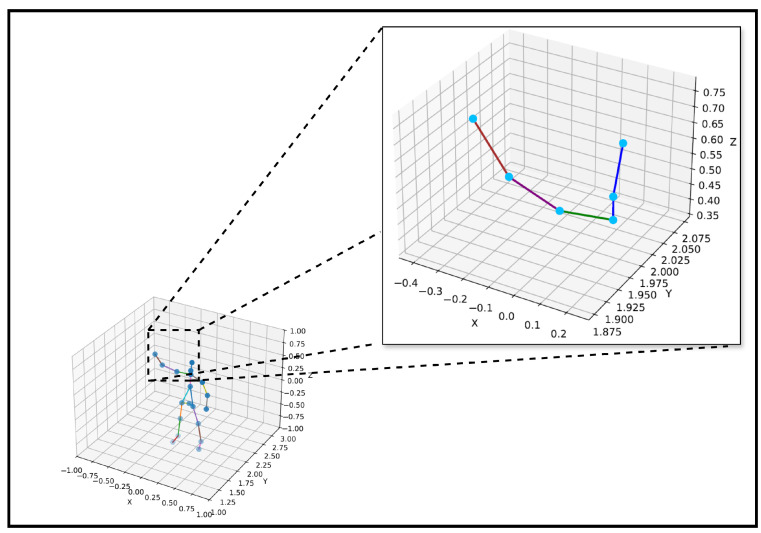
Visual inset skeleton for right upper limb.

**Figure 13:**
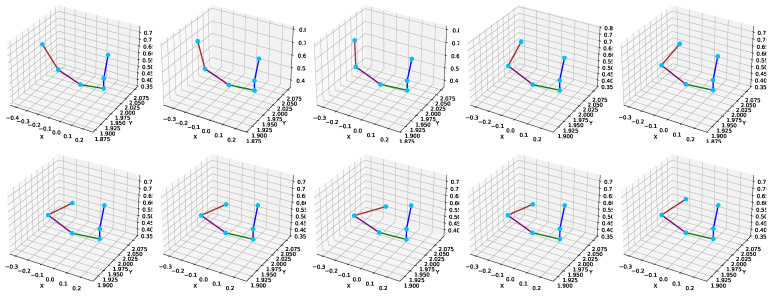
Time-series plots of joint positions showing model accuracy and consistency.

To complement the quantitative evaluation, Figure 12 and Figure 13 provide visual evidence of the model’s ability to maintain accurate and consistent joint estimations across time. Figure 12 presents a detailed inset visualization of the predicted right upper limb skeleton over multiple frames, showing the spatial continuity of joints, such as the right shoulder, right elbow, and right wrist. The skeleton shape is preserved across frames, demonstrating the model’s spatial stability and robustness under temporal variation.

Different colors are used to indicate specific joint connections to enhance the interpretability of the visualization in Figure 12. The blue lines represent the connections from the neck to the head, spine, and shoulder. The green line connects the spine shoulder to the right shoulder, the purple line denotes the connection from the right shoulder to the right elbow, and the brown line connects the right elbow to the right wrist. The joints are marked as cyan dots to ensure clear visibility of their spatial positions. Figure 13 uses a similar color-coding scheme to maintain visual consistency across visualizations.

In addition, Figure 13 displays time-series plots of joint coordinates corresponding to key upper body joints, including the right wrist, right elbow, right shoulder, neck, and head. These plots compare the predicted trajectories with ground-truth positions from the Kinect sensor. The curves exhibit smooth and consistent movement patterns that closely follow the ground truth. This visual alignment supports the temporal reliability of the proposed model, particularly in capturing complex articulations of the right upper limb.

To provide a more detailed understanding of model behavior across different types of motion, Table 18 presents the joint MAE and RMSE values for four representative activities: left upper limb extension (LUL), right side lung (RSL), right front lung (RFL), and left side lung (LSL). These activities were selected to reflect diverse body parts’ engagements, including unilateral upper limb movement and dynamic lower limb motion. The results indicate that the model achieves lower localization errors for the proximal joints, such as the base of the spine and the mid of the spine, in all activities. In contrast, distal joints, particularly the wrists and ankles, tend to exhibit higher errors, especially during complex motions, such as RFL and LSL. For example, the right ankle shows an MAE exceeding 2.0 cm in the right front lung, suggesting greater difficulty regarding estimation during low-limb activity.

Table 19 summarizes the average MAE and RMSE values across all the joints for each of the ten activities in the dataset. The results show variability in estimation performance across different activities, with Activity 1 yielding the lowest average error (MAE = 0.91 cm; RMSE = 1.05 cm), while Activities 4, 5, and 6 produce higher errors (MAE > 2.0 cm). These results concisely overview the model’s performance consistency across various movement types and highlight specific activity cases where joint estimation is more challenging.

### 4.3. Discussion

This research presents a novel framework for detecting human joint positions that provides benefits in accuracy and computational complexity. The combination of CNN, transformer, and Bi-LSTM components in one workflow shows synergy in effectively extracting spatial and temporal features.

To overcome the inherent shortcomings of mmWave radar data, namely sparsity and lack of consistency, the concatenation of adjacent frames plays a key role in enriching the point cloud data. By concatenating frames into a new and more informative frame, the model improves the quality of feature extraction in both the spatial and temporal domains. As demonstrated by the stable learning curve in n=4, this approach improves the detection accuracy and accelerates the model convergence. These results validate that this preprocessing step reduces noise artifacts and enhances signal relevance.

We split the processing into local and global processing. Global processing provides a broader understanding of the patterns and relationships across the entire dataset, which is important for understanding context and long-term dependencies and ensuring accurate predictions of joint positions. Transformer and Bi-LSTM blocks capture temporal data dependencies at different scales. This dual temporal processing flow ensures accurate joint position estimation, even in complex motion scenarios.

From several configurations, n=4 was selected as the optimal choice among the tested models as it offers good balance between feature complexity and computational load.

This research demonstrates significant performance improvements compared to conventional models such as MARS [3] and mmPose [17]. Unlike Shi et al. [1], who employed a multi-frame fusion approach to enhance point cloud density, our method concatenates adjacent frames to directly build a temporally and spatially more informative representation. By integrating a CNN, transformer, and Bi-LSTM, our model effectively captures spatiotemporal dependencies and robustly handles sparse radar point clouds. Specifically, our approach reduces the MAE by an average of 4.1 cm compared to the method proposed in [3]. It shows superior generalization by achieving lower RMSE values for training and validation sets.

This advantage is also proven through the paired *t*-test (Table 9 and Table 10), which shows that the difference in results between the proposed model and MARS [3] and mmPose [17] is statistically significant (*p* < 0.05). Although the comparison with CNN+Bi-LSTM [1] and mRI [15] is not statistically significant (*p* > 0.05), our model results still show lower error and better result stability. Even when compared with the quite competitive CNN+Bi-LSTM, our model still shows clear accuracy improvement and result stability, as evidenced by the 95% confidence intervals in Table 11, namely MAE ± CI = 1.77 ± 0.40 cm and RMSE ± CI = 2.91 ± 1.16 cm.

Furthermore, although the *p*-value for mRI [15] approaches the significance threshold (*p* ≈0.07), the observed absolute difference in MAE (4.26 cm vs. 1.77 cm) reflects a moderate to large effect size. This suggests that our model’s improvement is statistically relevant in some instances and practically meaningful for high-precision applications such as rehabilitation or clinical assessment.

In terms of computational efficiency, the inference time analysis in Table 12 shows that the proposed model has an average inference time of 0.28 ms per sample, which is higher than MARS [3] and CNN+Bi-LSTM [1]. This inference time increases due to the complexity of the architecture, especially the use of transformers and the frame merging process.

While the proposed model introduces additional architectural complexity, its average inference time remains practical for real-time use. As shown in Table 12, the model achieves an inference time of 0.28 ms per sample, which is well below typical real-time thresholds (e.g., 30–50 ms for visual feedback systems). Additionally, deployment on resource-constrained edge devices could benefit from standard optimization techniques, such as model pruning, quantization, and architectural simplification, to reduce the memory and compute demands without compromising real-time performance. The MARS model [3] was previously shown to operate with a latency of 64–105 μs per frame. Given that our model still provides substantial timing margin relative to sensor acquisition rates such as Kinect V2 (30 Hz = 33.3 ms) or mmWave radar (10 Hz = 100 ms), the observed accuracy improvement is justified by the acceptable increase in inference latency. Therefore, our model remains suitable for the real-time rehabilitation and deployment of monitoring systems.

In addition, to evaluate the generalization ability, the model was tested using the LOSO method, where one subject was used for testing and three others for training. The results from this scenario show that the model maintains its accuracy even when faced with data from subjects it has never seen before.

Apart from testing generalization using the LOSO approach, this research also evaluates the model’s ability to deal with more complex scenarios using the LSAO approach. The experimental results using the LSAO approach show that, despite a slight decrease in accuracy compared to the LOSO scenario, the proposed model still shows good robustness to changes in activity distribution. The decreases in MAE and RMSE are not too drastic, indicating that the model architecture, especially the frame concatenation and the application of transformer and Bi-LSTM, plays an important role in maintaining stable performance even when new activities are introduced during testing.

Although the MARS dataset primarily consists of dynamic rehabilitation movements, static human poses are inherently present within the sequences. These occur during natural pauses between transitions from one motion to another, such as when a subject temporarily stands still or holds a position before continuing to the next activity. As a result, the model is implicitly exposed to static pose patterns during training and evaluation. In these static intervals, frame-to-frame variation is minimal, and the concatenation of adjacent frames yields denser and more stable point clouds. This reduced temporal fluctuation likely enhances the accuracy of spatial feature extraction and joint localization, even in the absence of motion. Therefore, while not explicitly evaluated as a separate category, the model can generalize to static pose scenarios based on its exposure to such conditions within the dataset.

While the evaluation of the MARS dataset demonstrates strong performance and robustness, we acknowledge that testing on a single dataset may limit generalizability. As part of future work, we plan to collect a new radar dataset under diverse environmental settings and hardware configurations. This will allow for cross-dataset validation and the opportunity to assess the ecological validity of the proposed model in real-world deployment scenarios.

Moreover, as shown in Table 17, the ablation study further highlights the complementary importance of each network block in the proposed model. Removing the CNN block most significantly increases the error, emphasizing its role in early spatial feature extraction. The exclusion of the transformer or Bi-LSTM blocks also leads to notable performance degradation, confirming their roles in capturing temporal dependencies through attention and recurrent mechanisms. These results validate our choice of integrating a CNN, transformer, and Bi-LSTM to achieve a robust spatiotemporal representation.

To further support the temporal robustness of the proposed model, Figure 11 provides a visual sequence comparison where the predicted skeletons are consistently aligned with the Kinect-based ground truth across multiple frames. This visual coherence highlights the model’s ability to preserve joint structure over time. Additionally, Figure 13 presents time-series plots of joint trajectories, such as wrist and elbow, which exhibit smooth and continuous patterns closely following the ground truth. These visualizations collectively demonstrate the model’s temporal stability and reinforce the quantitative improvements in Table 6 and Table 18.

In addition to the quantitative evaluation, Table 18 and Table 19 provide more fine-grained insights by reporting the per-joint and per-activity performance. It is evident that wrist joints, which are located farther from the torso and exhibit greater motion, tend to have higher estimation errors. This trend is especially prominent in activities involving fast upper limb movements. These findings indicate that distal joints remain more challenging to estimate due to motion-induced sparsity and orientation variance, which could be a future target for improvement.

This can be explained by the fact that frame concatenation creates a richer temporal representation, while a transformer allows more flexible generalization of spatial patterns, and Bi-LSTM enhances the modeling of long-term temporal dynamics. The combination of these three blocks produces a latent representation that is more abstract and less dependent on one specific activity type, so the model can still recognize human movement patterns, even in activities that were not previously recognized during training.

A critical comparison shows that the conventional methods often focus on spatial or temporal modeling but lack integration of both. For instance, CNN-based approaches [1] effectively capture spatial features but cannot deal with temporal inconsistencies. In contrast, transformer-based models [28] are capable of temporal modeling but struggle with noise in sparse data. Our framework addresses these limitations by combining local and global processing, which allows our model to achieve state-of-the art results in radar-based human joint estimation.

Despite its benefits, the proposed approach faces some limitations:

1. Sensitivity to sparse data: Although merging adjacent frames effectively improves data quality, the sparseness of FMCW radar data still affects the accuracy of detecting difficult joint positions, such as the wrist and ankle. This limitation is evident from their higher MAE values compared to other joints.
2. Computational demand: Since merging adjacent frames produces a new one, increasing frame size demands more memory and computational resources, making real-time implementation challenging in resource-constrained environments.
3. Data diversity: Reliance on a single dataset such as MARS [3] limits the generalizability of the findings. Expanding the coverage of datasets for different human activities and environmental conditions is needed to validate the robustness of the model.

To address these challenges and build on the current findings, future research should explore the following directions:

1. Dynamic frame selection mechanisms: Introduce an adaptive frame selection model that dynamically selects frames based on motion intensity to further improve input quality while reducing unnecessary computational overhead.
2. Real-Time implementation: Optimizing real-time processing models is essential for practical applications. Techniques such as lightweight neural architectures, model pruning, and quantization can reduce the computational footprint without sacrificing accuracy.
3. Edge-level deployment feasibility: To support future deployment, the proposed model is designed with a radar-compatible architecture that can be optimized for edge devices such as the TI IWR1443. Although real-time benchmarking is not included in this work, the model’s modular structure enables the potential use of lightweight techniques, such as pruning or quantization, to reduce latency and computational cost. This direction is promising for practical applications like in-home rehabilitation and fall detection.
4. Generalization across datasets: Future research should focus on training and validating the model on multiple datasets with varying environmental conditions and movement patterns. While the evaluation using the MARS dataset [3] has demonstrated strong performance, testing on a single dataset may limit the external validity of the findings. As part of future work, we plan to collect a new radar dataset under diverse environmental settings and hardware configurations. This will allow for cross-dataset validation and the opportunity to assess the ecological validity of the proposed model in real-world deployment scenarios.

## 5. Conclusions

In this paper, we introduced a novel approach for human skeleton estimation by leveraging the combination of adjacent frames to create a richer and more comprehensive representation of input data, thereby improving feature quality and model performance. By integrating CNN, transformer, and Bi-LSTM architectures, the proposed model effectively captures spatial and temporal patterns, significantly reducing estimation error compared to conventional models. The experimental results demonstrate that our method of concatenating frames leads to more informative input data, allowing the model to perform well in predicting human joint positions. This research advances the understanding of spatiotemporal dynamics in human skeleton estimation. It sets a new benchmark in the field by outperforming established methods regarding accuracy and computational efficiency.

The experimental setup is designed to align with methodologies established in previous research, ensuring the reliability and robustness of the results. This research provides a comprehensive evaluation of the proposed model’s capabilities by utilizing the MARS public dataset and implementing a series of controlled experiments. The findings highlight the advantages of the frame concatenation approach, as evidenced by the superior performance of our model for n=4, which achieved the best balance between capturing complex features and maintaining computational efficiency. This model demonstrates a marked improvement over other configurations, emphasizing the importance of optimizing the frame selection process to enhance model performance.

Furthermore, the ablation study reaffirms the necessity of including all three components in the model. The removal of any one of the CNN, transformer, or Bi-LSTM modules leads to performance drops, validating their synergistic contribution to accurate joint estimation. Additional evaluations across joints and activities also reveal that wrist joints pose higher estimation challenges, particularly in dynamic-motion scenarios, providing important direction for future model refinement.

The combination of CNN, transformer, and Bi-LSTM blocks, alongside the use of frame concatenation, results in a robust and efficient model that significantly outperforms the conventional approaches.

## Figures and Tables

**Table 1:** The architecture of neural network for experiment.

Layer Type	Number of Filters	Filter Size
Conv2D	32	3 × 3
MaxPooling2D		2 × 2
Conv2D	128	3 × 3
MaxPooling2D		2 × 2
Conv2D	64	3 × 3
MaxPooling2D		2 × 2
Multi-Head Attention	8	
Bi-LSTM	128	
Dense	72	

**Table 2:** Configuration in the experiment.

Name	Information
GPU	NVIDIA GeForce RTX 4090 24 GB
Radar Model	TI IWR1443
Frequency Range	76–81 GHz
Number of TX Antennas	3
Number of RX Antennas	4
Number of Virtual Antennas	12 (via MIMO)
Chirp Bandwidth	3.2 GHz
Chirp Duration	32 μs
Range Resolution	4.69 cm
Velocity Resolution	0.35 m/s
Angular Resolution	9.55°
Operation System	Windows 11
Simulation Environment	Python 3.10.3; keras 2.9.0; tensorflow 2.9.1

**Table 3:** Training parameters.

Parameters	Description
Adam	Optimizer
Epochs	150
Learning Rate	0.001
Batch Size	128

**Table 4:** A comparison of the average MAE and RMSE values for 19 human joint positions across different values of *n*.

	*x* (cm)			*y* (cm)			*x* (cm)			Average (cm)	
Model	MAE	RMSE		MAE	RMSE		MAE	RMSE		MAE	RMSE
n=1	2.13	3.72		1.75	2.62		1.97	3.51		1.95	3.29
n=2	1.99	3.40		1.66	2.46		1.80	3.17		1.82	3.01
n=3	2.00	3.42		1.65	2.44		1.79	3.11		1.81	2.99
n=4	**1.94**	**3.28**		**1.62**	**2.39**		**1.76**	**3.07**		**1.77**	**2.92**
n=5	1.95	3.27		1.63	2.43		1.77	3.12		1.78	2.94

**Note:** Bold indicates the best performance among the configurations.

**Table 5:** Comparison of the total number of parameters for 19 human joint positions under varying values of *n*.

Model	Total Parameters
*n* = 1	353,609
*n* = 2	649,673
*n* = 3	1,109,577
*n* = 4	1,733,321
*n* = 5	2,106,633

**Table 6:** The experimental results for the average MAE and RMSE for n=4.

	*x* (cm)			*y* (cm)			*x* (cm)			Average (cm)	
Joints	MAE	RMSE		MAE	RMSE		MAE	RMSE		MAE	RMSE
Spine-Base	1.47	2.59		1.23	1.88		1.29	2.36		1.33	2.28
Spine-Mid	1.56	2.76		1.14	1.63		1.35	2.64		1.35	2.34
Neck	1.91	3.23		1.50	2.08		1.52	2.97		1.64	2.76
Head	2.17	3.56		1.74	2.40		1.64	3.20		1.85	3.05
Left Shoulder	1.90	3.21		1.55	2.11		1.59	2.82		1.68	2.71
Left Elbow	2.33	3.57		1.84	2.58		2.72	4.13		2.30	3.43
Left Wrist	3.04	4.87		2.30	3.18		3.73	6.30		3.02	4.78
Right Shoulder	1.87	3.15		1.59	2.25		1.68	2.90		1.71	2.77
Right Elbow	2.29	3.57		2.01	2.89		2.62	3.92		2.30	3.46
Right Wrist	3.02	4.82		2.45	3.34		3.54	5.89		3.00	4.69
Left Hip	1.48	2.59		1.26	1.89		1.27	2.31		1.33	2.26
Left Knee	1.60	2.78		1.63	2.50		1.38	2.18		1.54	2.48
Left Ankle	1.83	3.41		1.39	2.43		1.13	2.36		1.45	2.73
Left Foot	2.00	3.67		1.84	3.06		1.39	2.72		1.74	3.15
Right Hip	1.48	2.55		1.28	1.95		1.30	2.35		1.35	2.28
Right Knee	1.60	2.68		1.62	2.43		1.40	2.10		1.54	2.40
Right Ankle	1.65	2.97		1.30	2.10		1.10	2.00		1.35	2.36
Right Foot	1.86	3.28		1.78	2.79		1.33	2.32		1.66	2.80
Spine-Shoulder	1.81	3.10		1.37	1.91		1.47	2.89		1.55	2.63
**Average**	**1.94**	**3.28**		**1.62**	**2.39**		**1.76**	**3.07**		**1.77**	**2.92**

Note: “Average” refers to the mean MAE and RMSE across 19 joints in the experiment where *n* = 4.

**Table 7:** Comparison of MAE and RMSE values from the estimation of 19 connections based on the experimental results of several different models.

	*x* (cm)			*y* (cm)			*z* (cm)			Average (cm)	
Model	MAE	RMSE		MAE	RMSE		MAE	RMSE		MAE	RMSE
MARS [3]	6.99	9.97		4.07	5.56		6.54	8.94		5.87	8.10
CNN+Bi-LSTM [1]	2.22	3.62		1.63	2.42		1.87	3.22		1.91	3.09
mmPose [17]	6.80	10.21		4.79	6.67		6.94	9.86		6.18	8.91
mRI [15]	5.08	7.52		2.71	3.73		5.00	7.10		4.26	6.12
**Proposed method**	**1.94**	**3.28**		**1.62**	**2.39**		**1.76**	**3.07**		**1.77**	**2.92**

**Note:** Bold values indicate the best results obtained with the proposed configuration (*n* = 4).

**Table 8:** Parameter comparison across models for estimating 19 human joints.

Model	Total Parameters
MARS [3]	1,095,115
CNN+Bi-LSTM [1]	799,465
mmPose [17]	2,281,739
mRI [15]	3,247,481
**Proposed method**	**1,733,321**

**Note:** Bold values indicate the best results obtained with the proposed configuration (*n* = 4).

**Table 9:** MAE-based statistical comparison using *t*-test and *p*-value.

Model	*t*-Statistic	*p*-Value
MARS [3]	4.95	0.038
CNN+Bi-LSTM [1]	1.69	0.232
mmPose [17]	7.06	0.019
mRI [15]	3.55	0.070

**Table 10:** The *t*-test and *p*-value analysis for RMSE comparison between models.

Model	*t*-Statistic	*p*-Value
MARS [3]	4.93	0.038
CNN+Bi-LSTM [1]	1.92	0.194
mmPose [17]	6.96	0.019
mRI [15]	3.43	0.075

**Table 11:** Confidence interval analysis across conventional and proposed models.

Model	MAE ± 95%∼CI (cm)	RMSE ± 95%∼CI (cm)
MARS [3]	5.87±3.91	8.16±5.73
CNN+Bi-LSTM [1]	1.91 ± 0.74	3.09 ± 1.52
mmPose [17]	6.18 ± 2.99	8.91 ± 4.85
mRI [15]	4.26 ± 3.34	6.12 ± 5.16
Proposed model	1.77 ± 0.40	2.91 ± 1.16

**Table 12:** Performance comparison in terms of inference time and accuracy across models.

Model	Average Inference Time per Sample (ms)	Accuracy (%)
MARS [3]	0.03	96.44
CNN+Bi-LSTM [1]	0.13	98.55
Proposed model	0.28	98.77

**Table 13:** Performance of the MARS model on unseen subjects using LOSO testing.

Unseen Subject			*x* (cm)			*y* (cm)			*z* (cm)			Average (cm)	
Training	Test		MAE	RMSE		MAE	RMSE		MAE	RMSE		MAE	RMSE
Subj_1, 2, 3	Subj_4		9.09	11.42		10.07	13.06		6.59	8.63		8.58	11.04
Subj_1, 2, 4	Subj_3		6.12	8.38		7.86	10.51		5.45	7.49		6.48	8.79
Subj_1, 3, 4	Subj_2		11.11	13.69		10.76	13.7		7.43	10.44		9.76	12.61
Subj_2, 3, 4	Subj_1		8.12	10.80		9.54	12.52		6.26	8.71		7.97	10.68

**Table 14:** MAE and RMSE results of CNN+Bi-LSTM across unseen subjects under LOSO testing.

Unseen Subject			*x* (cm)			*y* (cm)			*z* (cm)			Average (cm)	
Training	Test		MAE	RMSE		MAE	RMSE		MAE	RMSE		MAE	RMSE
Subj_1, 2, 3	Subj_4		2.19	3.12		4.66	6.33		5.65	6.85		4.17	5.43
Subj_1, 2, 4	Subj_3		1.85	2.60		3.71	5.16		4.35	5.88		3.30	4.55
Subj_1, 3, 4	Subj_2		2.87	3.59		4.94	7.05		7.22	9.34		5.01	6.66
Subj_2, 3, 4	Subj_1		2.30	3.13		5.15	7.22		5.88	7.66		4.44	6.00

**Table 15:** Performance of the proposed model (*n* = 4) on unseen subjects using LOSO evaluation.

Unseen Subject			*x* (cm)			*y* (cm)			*z* (cm)			Average (cm)	
Training	Test		MAE	RMSE		MAE	RMSE		MAE	RMSE		MAE	RMSE
Subj_1, 2, 3	Subj_4		2.11	2.91		4.63	6.23		5.66	6.84		4.13	5.32
Subj_1, 2, 4	Subj_3		0.77	1.19		1.59	2.56		1.44	2.43		1.26	2.06
Subj_1, 3, 4	Subj_2		2.96	3.67		5.38	7.63		7.32	9.56		5.21	6.96
Subj_2, 3, 4	Subj_1		2.26	3.05		5.16	7.17		5.82	7.49		4.42	5.90

**Table 16:** Evaluation results of the proposed method under LSAO setup across five rounds.

		*x* (cm)			*y* (cm)			*z* (cm)			Average (cm)	
Round		MAE	RMSE		MAE	RMSE		MAE	RMSE		MAE	RMSE
Round 1		1.76	2.22		7.71	9.76		3.60	4.60		4.36	5.53
Round 2		1.47	1.84		9.08	11.19		1.82	2.53		4.12	5.19
Round 3		1.34	1.73		6.90	9.12		2.14	3.01		3.46	4.62
Round 4		1.45	1.81		8.33	10.39		1.98	2.72		3.92	4.97
Round 5		1.62	1.98		8.89	10.85		2.32	3.12		4.28	5.32

**Table 17:** Ablation study results when removing major components.

Model	*x* (cm)			*y* (cm)			*z* (cm)			Average (cm)	
	MAE	RMSE		MAE	RMSE		MAE	RMSE		MAE	RMSE
CNN + Trans. (–Bi-LSTM)	2.12	3.57		2.84	3.50		1.93	3.13		2.30	3.40
CNN + Bi-LSTM (–Trans.)	2.31	3.86		1.78	2.64		2.13	3.53		2.07	3.34
Trans. + Bi-LSTM (–CNN)	4.34	7.16		2.79	4.04		3.85	6.08		3.66	5.76
CNN + Trans. + GRU (–Bi-LSTM)	2.02	3.38		1.70	2.49		1.79	3.07		1.84	2.98
**Proposed Model**	**1.94**	**3.28**		**1.62**	**2.39**		**1.76**	**3.07**		**1.77**	**2.92**

**Note:** Bold values highlight the best performance achieved by our model (*n* = 4).

**Table 18:** MAE and RMSE for each joint across different activities.

Joints	LUL			RSL			RFL			LSL	
	MAE	RMSE		MAE	RMSE		MAE	RMSE		MAE	RMSE
Spine-Base	0.81	0.84		0.76	0.79		1.12	1.19		1.69	1.72
Spine-Mid	0.69	0.83		1.29	1.51		1.42	1.58		1.94	2.08
Neck	1.68	2.38		1.59	2.01		2.42	2.96		2.32	2.60
Head	0.93	1.09		1.33	1.76		2.49	2.96		2.36	2.66
Left Shoulder	0.65	0.69		0.77	0.86		2.32	2.53		2.61	2.86
Left Elbow	1.03	1.28		1.39	1.63		1.93	2.41		2.37	2.74
Left Wrist	0.85	0.90		0.88	1.01		1.24	1.43		1.66	1.83
Right Shoulder	1.21	1.33		1.01	1.10		2.15	2.33		1.54	1.69
Right Elbow	1.33	1.54		1.64	2.19		2.28	2.91		1.63	1.89
Right Wrist	0.85	0.97		1.76	2.06		2.02	2.31		2.83	3.04
Left Hip	0.82	0.94		0.88	1.02		2.45	2.73		2.25	2.49
Left Knee	0.61	0.70		0.88	1.02		2.23	2.65		2.22	2.41
Left Ankle	0.71	0.82		0.98	1.13		2.23	2.60		1.87	2.22
Left Foot	0.77	0.81		0.51	0.70		1.55	2.23		1.43	1.90
Right Hip	1.87	2.27		3.07	3.70		2.12	2.42		3.17	3.84
Right Knee	1.01	1.15		0.63	0.82		1.97	2.28		2.27	2.72
Right Ankle	0.55	0.65		0.46	0.57		1.66	1.99		1.32	1.44
Right Foot	0.60	0.69		0.83	1.03		1.73	2.04		1.84	2.01
Spine-Shoulder	0.45	0.52		0.46	0.58		1.43	1.77		1.53	1.74
Average	0.91	1.05		1.23	1.44		2.00	2.28		2.06	2.31

**Table 19:** Average MAE and RMSE for each activity.

Activity	Average (cm)	
	MAE	RMSE
Activity 1	0.91	1.05
Activity 2	1.61	1.82
Activity 3	2.08	2.34
Activity 4	2.10	2.40
Activity 5	2.00	2.28
Activity 6	2.06	2.39
Activity 7	1.23	1.44
Activity 8	2.06	2.31
Activity 9	1.68	1.89
Activity 10	1.42	1.65

## Data Availability

This work uses simulated data created using the simulation software introduced in Ref. [3].

## Abbreviations

The following abbreviations are used in this manuscript:

Bi-LSTM	Bidirectional Long Short-Term Memory
Bi-GRU	Bidirectional Gated Recurrent Unit
CI	Confidence Interval
CNN	Convolutional Neural Network
FFN	Feed-Forward Network

FMCW	Frequency-Modulated Continuous Wave
GCN	Graph Convolutional Network
GNN	Graph Neural Network
GPU	Graphics Processing Unit
GRU	Gated Recurrent Unit
HAR	Human Activity Recognition
ISAR	Inverse Synthetic Aperture Radar
LiDAR	Light Detection And Ranging
LOSO	Leave One Subject Out
LSAO	Leave Some Activities Out
MAE	Mean Absolute Error
mmWave	Millimeter Wave
NLP	Natural Language Processing
RMSE	Root Mean Squared Error
